# Measuring productivity and its relationship to community health worker performance in Uganda: a cross-sectional study

**DOI:** 10.1186/s12913-018-3131-9

**Published:** 2018-05-09

**Authors:** Naoko Kozuki, Tana Wuliji

**Affiliations:** 1 Department of International Health, Johns Hopkins Bloomberg School of Public Health, Baltimore, MD USA; 2 University Research Co., Bethesda, MD USA

**Keywords:** Health worker performance, Health worker productivity, CHW, CHV, Uganda

## Abstract

**Background:**

To explore the nature of the relationship between and factors associated with productivity and performance among the community health volunteer (CHV) cadre (Village Health Teams, VHT) in Busia District, Eastern Uganda. The study was carried out to contribute to the global evidence on strategies to improve CHV productivity and performance.

**Methods:**

This cross-sectional study was conducted with 140 VHT members as subjects and respondents. Data were collected between March and May 2013 on the performance and productivity of VHT members related to village visits and activities for saving maternal and child lives, as well as on independent factors that may be associated with these measures. Data were collected through direct observation of VHT activities, structured interviews with VHTs, and review of available records. The correlation between performance and productivity scores was estimated, and LASSO regression analyses were conducted to identify factors associated with these two scores independently.

**Results:**

VHTs demonstrated wide variation in productivity measures, conducting a median of 13.2 service units in a three-month span (range: 2.0-114.9). Performance of the studied VHTs was generally high, with a median performance score (out of 100) of 96.4 (range: 50.9-100.0). We observed a weak correlation coefficient of 0.05 (*p* = 0.57) between productivity and performance scores. Older VHT age (≥50 years old, reference: <50 years old) (11.14, 95% CI: 3.26-19.01) and knowledge of danger signs (in units of ten-percentage points, 1.92, 95% CI: 0.01-3.83) were positively associated with productivity scores. Job satisfaction (1.46, 95% CI: 0.13-2.80) and knowledge of danger signs (in units of ten-percentage points, 1.02, 95% CI: 0.05-1.98) were positively associated with performance scores.

**Conclusions:**

Older VHT age and knowledge of danger signs were positively associated with productivity, and job satisfaction and knowledge of danger signs were positively associated with performance. No correlation was observed between productivity and performance scores. This lack of correlation suggests that interventions to improve CHV effectiveness may affect the two dimensions of effectiveness differently. We recommend that productivity and performance both be monitored to evaluate the overall impact of interventions to increase CHV effectiveness.

**Electronic supplementary material:**

The online version of this article (10.1186/s12913-018-3131-9) contains supplementary material, which is available to authorized users.

## Background

Inspired by the Alma Ata health care for all movement, community health worker (CHW) / community health volunteer (CHV) programs have been established in many countries to empower communities to reduce preventable mortality and morbidity. A number of studies and high-level forums have recognized the essential contributions that CHVs and CHWs make and their impact on the health of the community [1–4]. CHW and CHV effectiveness is affected by how well they do their work against expected standards (performance), how much they can feasibly do (productivity), and what factors affect their work [5]. Performance is defined in the literature in a multitude of ways, and some definitions incorporate the element of productivity. For our purposes, performance is defined as the extent to which tasks or services are completed according to standards, as a measure of how well tasks are done; whilst productivity is defined as the volume of services, or how much is done. A better understanding of these factors will allow the greater health community to deploy and support them to maximize their effectiveness.

The expectations of CHWs and CHVs have increased over time, with many countries expanding their scope of practice and responsibilities, potentially overburdening them [6]. CHW and CHV performance has been studied extensively [5, 7, 8], but fewer studies have examined their productivity [5, 9]. To our knowledge, the interplay of factors that can affect both productivity and performance has not been examined in detail.

In the year 2000, the Ugandan Ministry of Health (MoH) established Village Health Teams (VHT) to empower communities to take part in the decisions that affect their health, to mobilize communities for health programs, and to strengthen the delivery of health services at the household level. VHTs are unsalaried CHVs who are linked to the lowest level of the health care system, or Health Center I. The MoH reports the following guidelines for VHT selection: must be 18 years of age or older, reside in the village, literate in the local language, capable of community mobilization and communication, dependable, trustworthy, interested in health and development, and willing to work for the community [10]. The MoH recommendation is that one male and one female community member be selected through popular vote of stakeholders and households in the village to constitute the VHT, though in larger communities, more than two VHT members may be chosen.

A VHT is responsible for approximately 30 households [11]. VHT responsibilities are delineated into seven categories: record (collecting data on catchment population), model (practicing model health behaviour), save lives (providing health services and referrals), link (improving linkages between formal health care and their catchment areas), mobilize (mobilizing village members and health workers for health activities), visit (visiting households and communities), and support (assisting in care of those living with illness / HIV) [12]. More details of their responsibilities are available in Additional file 1. In recent years, VHTs have been increasingly tasked with strengthening community support for people living with HIV, including tasks such as mapping HIV patients in the community; linking patients to VHTs and other resources; engaging community groups to support HIV patients to address their needs such as nutrition, psychosocial support, savings, and loan; and supporting patient self-care through health goal setting to improve health status [13]. Several studies have demonstrated the importance of VHTs in Uganda and their potential to further improve health services and outcomes [14, 15].

This study seeks to describe VHT productivity and performance in Busia District, Uganda, explore the relationship between VHT productivity and performance, and investigate independent factors that influence VHT productivity and performance. The study was conceived to contribute to the global evidence on strategies to improve CHW/CHV productivity and performance with an eye toward better understanding factors that could increase their effectiveness.

## Methods

### Study design

We conducted a cross-sectional study to assess levels of VHT productivity and performance in Busia District, Eastern Uganda between March and May 2013. Training of the VHTs was conducted by the MoH in collaboration with World Vision International using the standard training guide for VHTs developed by the MoH. Training length varied though the same training package and facilitators guide was used. Specific details of training undertaken by each VHT were not available. Supervisory systems varied across districts. In the district under study, the VHTs were supervised by World Vision International through quarterly review meetings that brought all the VHTs together.

To quantify productivity and performance, our study focused only on the “save lives” and “visit” tasks of the basic package of services as defined by the Ugandan MoH (full description of the six components of the basic package of services can be found in Additional file 1). The study population included all VHTs who provided the basic package of services in two randomly selected sub-counties out of a total of ten sub-counties in Busia District. Data were collected through multiple sources. In one, the data collector observed a VHT assess a sick child (2-59 months) and a pregnant mother for danger signs during a home visit. The VHT recorded his/her findings, illness classification, and management on an observation checklist. Immediately after the consultation, the data collector conducted a comparative, “gold standard” clinical evaluation of the child to determine 1) if the VHT had missed any danger signs that required immediate attention or referral and 2) the extent to which the VHT’s evaluation was correct against the standard. During the same visit, the quality of interaction with the household and the length of time the home visit took, including travel time, were recorded as well. For quality of the interaction, a checklist was used by the data collectors that included points such as whether the VHT addressed the family politely and respectfully, whether relevant advice was given, and whether the VHT facilitated agreement with the family on what actions should be taken by whom and when. Data collectors were instructed to build rapport with the observed VHTs to minimize any issues. Due to resource limitations and logistical constraints, only one direct observation was conducted per VHT.

The second data source was routine data already being collected by VHTs, such as village maps, village registers, and monthly summary forms. Monthly data were taken from August to October 2013. These were reviewed for completeness and to quantify the frequency of activities conducted by the VHTs. The frequency data were taken from VHT registers and monthly summary forms. Finally, surveys were administered to VHTs and supervising health facility staff to collect data on individual characteristics of the VHTs. The variables chosen for collection were identified from the literature and also suggested by stakeholders in a November 2012 consultation held in Kampala, Uganda, and grouped into categories (Additional file 1). Data were triangulated where possible to address discrepancies, and some data were excluded because they formed patterns of reporting that were not feasible. Occasionally, frequency of a particular intervention was not documented, thus self-report was used. This was then verified with those interviewed in the village to ensure that these were as accurate as possible.

Data collection tools were translated from English into the local language in Busia, independently reviewed and revised, and back-translated into English for comparison. They are available on Additional file 2. Data collection tools were piloted in a sub-county not selected for the study. Data were entered into a proforma template in EpiInfo. Data regarding reported frequency of activities were cross-checked with standard documentation such as monthly reports and VHT registers, where available. Data entry quality checks were completed for 10% of the data.

### Sample size

The following equation was used for sample size calculation: n = [DEFF*Np(1-p)]/ [(d^2^/Z^2^_1-α/2_*(N-1) + p*(1-p)] [14]. The sample size formula is standard for prevalence-based sampling. We used the VHT’s ability to correctly elicit and respond to danger signs as basis for the sample size calculation, using 67% (p) as reported in a 2011 study in the Eastern Districts of Uganda [14]. We assumed this percentage applied to Busia District. Note that there was no other comparable basis upon which to calculate the sample size. With a precision of 5% (d) with a 95% confidence interval (α of 0.05), and a design effect (DEFF) of one, we estimated that each sub-county needs a sample of 65 VHTs to detect the 67% prevalence.

#### Ethics, consent, and permission

Ethics approval was received from the Institutional Review Boards of University Research Co., LLC, Makerere College of Health Sciences, and the Uganda National Council for Science and Technology. Verbal informed consent was obtained from all participants.

#### Productivity and performance measures

##### Productivity

Productivity was measured as the total volume of output of a service provided by one VHT during the three months prior to the date of data collection. The study collected data on the frequency of completion of the activities/tasks related to visiting villages and assessing danger signs of children and pregnant mothers, and then aggregated them into a composite unit that represented a single volume of output. The middle column of Table 1 describes the variables used to derive the composite productivity score.

**Table 1:** Description of VHT productivity and performance measures

VHT service category	Description of productivity measures (for the past three months)	Description of performance measures
Visiting village members	1. The number of homes/families visited by VHT 2. Time taken to complete a typical home visit	Assessment of how well a home visit is conducted by a VHT, based on 1. Whether family members were receptive to the visit 2. Whether the purpose of the visit is explained to household members 3. Whether health behavior changes are agreed upon jointly with household members 4. Whether families agree to implement suggested change 5. Whether the VHT plans a follow-up visit to check if changes are made This was summarized into what percent of the five tasks a VHT successfully completed.
Helping save lives	Assessing danger signs in children and pregnant mothers 1. Number of children evaluated for danger signs 2. Number of pregnant mothers evaluated for danger signs 3. Time taken to complete activities	Percent agreement of the VHT and gold standard clinician in identifying danger signs in a child (< 5 years of age)
		Percent agreement of the VHT and gold standard clinician in identifying danger signs in a pregnant woman

We adapted a method used by the World Bank to create a Composite Services Index (CSI) [16], which is an aggregate measure of the quantity of health services delivered by health workers. CSI is expressed as service equivalents. For our purposes, we used the unit of “home visit” as the service to make the other services (evaluation of children and pregnant mothers) comparable to. The details on the derivation of CSI are available in Additional file 1. The CSI served as the productivity score.

##### Performance

Performance was calculated as a standardized score, representing the extent to which tasks were completed to expected standards. We collected data on the quality of interactions between VHT and families during home visits (task two of basic package of services) and on how accurately VHTs identified danger signs in pregnant women and children (task three of basic package of services). The right column of Table 1 describes the variables used to derive a performance score. Each cell of performance measures listed in the right column of Table 1 was standardized into a scale of 0-100%, averaged for each task group first, then averaged again to derive the performance score.

#### Data analysis

We calculated and summarized the productivity and performance scores and the independent variables (listed in Table 2) across VHTs. The correlation coefficient was calculated between the productivity and performance scores to describe the relationship between the two variables. We then ran regression analyses to estimate the associations between the independent variables and productivity and performance, respectively. Independent variables that we were able to collect complete data on and had enough variability are listed in Table 2 and were included as covariates in the regression models. For pre-defined groupings of independent variables, non-linear Principal Component Analysis (PCA) was conducted to reduce the number of variables. The first component, which explains the largest amount of variation among the selected variables, was retained as the summary measure for that set of variables [17, 18]. The categories that summary measures were created for include VHT’s job satisfaction, VHT’s belief that he/she is a change agent, VHT’s sense of accountability, and how VHTs perceived the quality of the supervision they received. Equipment availability was tabulated, out of twelve total pieces of equipment identified as essential or necessary for VHT activity (mid-upper arm circumference tape, VHT handbook, job aids, referral forms, summary forms, VHT register, VHT maps, respiratory timers, soap, clean water, thermometer, and information, education, and communication materials).

**Table 2:** Description of factors that may influence productivity and performance

Influencing factors	Description of independent variables
1. VHT	Sex
	Age (< 35, 35- < 50, ≥50)
	Marital status (married, single)
	Flooring at own residence (covered with cement, tiles, concrete, or carpet, not covered)
	Years of schooling
2. VHT engagement in their work and with other VHTs	Job satisfaction^a^ - Ratings on a five-point Likert-type scale on statements relating to being proud to be a VHT, feeling happy with the VHT work, ease of interaction with others, being known for their reliability as a VHT
	Change agent^a^ - Ratings on a five-point Likert-type scale on statements relating to helping other VHTs learn new skills, encouraging other VHTs to discuss challenges, applying new skills in their work, suggesting solutions, and giving feedback to other VHTs
	Accountability^a^ - Ratings on a five-point Likert-type scale on statements relating to seeing their VHT work through to completion, completing tasks on time, perception of clarity of goals, evaluating their own performance
3. VHT competencies	Received VHT basic services training
	Knowledge of danger signs in pregnant women, newborns, and children (summarized as percent correct for each of the three target populations, then averaged)
4. Incentives	Financial incentives received
5. Recognition	VHT perceived that health facility is supportive
6. Supervision	Frequency of supervision over last 3 months
	Perceived quality of supervision against VHT program supervision requirements^a^ – Checks equipment, provides feedback, finds solution to problems
7. Equipment and supplies	Availability of essential supplies required for provision of VHT services (e.g., soap, clean water)
	Ready access to transportation (motorcycle or bicycle)
	Availability of a cell phone
8. Client factors	For the child case observed - Age of child - Number of complaints described by caregiver during interactions
	For the mother counselling observed - Age of mother - Number of years of schooling completed by the mother

^a^Made into score using Principal Component Analysis

Multi-level multiple linear regression, accounting for clustering at the sub-county level, was conducted to identify variables that are statistically significantly associated with productivity and performance independently. Separately, LASSO regression [19] was also conducted; this statistical method takes into account the collinearity between variables and creates a more parsimonious model through variable selection. The selected variables were refitted in a separate multi-level multiple linear regression model to account for regression coefficients that are biased toward zero in the LASSO regression [20, 21]. Stata version 13.0 (StataCorp, College Station, TX) was used for the analysis. The data file is available upon request.

## Results

Data were collected for 147 VHTs available in these two districts, 79 from Sub-county 1 and 68 from Sub-county 2. Five VHTs had substantial missing data, and two VHTs had outlier quantities in key variables comprising the productivity variable. A final total of 140 (95.2% of total surveyed) VHTs were thus included in the analysis. Their background characteristics are described in Table 3. Of the 140 VHTs, 52% were female and 51% were between the ages of 35- < 50. The VHTs had an average of 9.3 years of education and an average age of 40.7. The loadings for the first component from the PCA-derived variables are available in Additional file 1.

**Table 3:** Characteristics of VHTs included in the study, % or mean (SD)

	Combined (*n* = 140)	Subcounty 1 (*n* = 77)	Subcounty 2 (*n* = 63)
Sex			
Male	47.9%	44.2%	52.4%
Female	52.1%	55.8%	47.6%
Age	40.7 (9.6)	41.2 (9.3)	40.1 (10.1)
< 35	27.9%	24.7%	31.8%
35- < 50	51.4%	54.6%	47.6%
> =50	20.7%	20.8%	20.6%
Married / in long-term relationship	92.3%	96.2%	87.3%
Have covered flooring in residence	76.1%	73.4%	79.4%
Years of education	9.3 (2.2)	9.2 (2.3)	9.3 (2.1)
Years serving as a VHT	3.1 (3.8)	3.8 (4.3)	2.2 (2.8)
Received basic VHT training	97.1%	98.4%	97.1%
Knowledge of danger signs (in percent)	54.7 (16.9)	54.4 (16.1)	55.1 (18.0)
Financial incentives received	91.4%	90.9%	92.1%
Perceived that health facility is supportive	91.4%	93.5%	88.9%
Frequency of supervision received over last 3 months	2.1 (1.2)	2.4 (1.2)	1.8 (1.1)
Availability of supplies (out of twelve)	9.7 (1.0)	9.6 (0.8)	9.8 (1.1)
Motorcycle or bicycle available	65.0%	71.4%	57.1%
Cell phone available	79.3%	75.3%	84.1%
Age of child examined (in months)	21.1 (13.8)	20.7 (12.8)	21.6 (15.1)
< 12 months	25.0%	22.1%	28.6%
≥ 12 months	75.0%	77.9%	71.4%
Number of child health complaints described by caregiver	2.2 (0.9)	2.0 (0.6)	2.3 (1.2)
Age of the mother whose child is being examined	25.0 (6.3)	25.4 (6.4)	24.6 (6.1)
< 18	6.4%	3.9%	9.5%
18-35	84.3%	85.7%	82.5%
≥ 35	9.3%	10.4%	7.9%
Years of schooling received by the mother whose child is being examined	6.5 (2.9)	6.4 (2.4)	6.6 (3.4)
< 7 years	60.7%	61.0%	60.3%
7- < 12 years	31.4%	29.9%	33.3%
≥ 12 years	7.9%	9.1%	6.4%

The productivity and performance scores are summarized in Table 4. CSI, with each unit an equivalent of a home visit, ranged widely. VHTs conducted a median of 13.2 and mean of 20.8 service units in a three-month span, with a range of 2.0-114.9. The performance score was generally high, with a mean of 91.0 and median of 96.4, with a range of 50.9-100.0. There was no evidence of either positive or negative correlation between productivity and performance, with a correlation coefficient of 0.05 (*p* = 0.57).

**Table 4:** Summary of productivity and performance measure

Outcome measure	Mean (SD)	Median	Range
Productivity			
Number of home visits made in last 90 days	10.2 (13.7)	6	1-90
Time taken to complete one home visit (in hours)	0.7 (0.4)	0.6	0.2-2.8
Number of children evaluated in last 90 days	4.2 (5.5)	3	0-41
Time take to evaluate one child (in hours)	0.8 (0.4)	0.7	0.2-3.2
Number of mothers evaluated in last 90 days	3.8	3	0-30
Time taken to evaluate one mother (in hours)	0.8 (0.4)	0.7	0.2-2.0
Productivity score (Composite Service Index, unit standardized to a home visit)	20.8 (20.6)	13.2	2-114.9
Performance			
% of home visit performance measures completed (Table 3)	90% (19%)	100%	20-100%
Percent agreement between VHT and clinician of child danger signs	95% (10%)	100%	56-100%
Percent agreement between VHT and clinician of pregnant woman danger signs	89% (16%)	100%	14-100%
Performance score (scale of 0-100)	91.0 (11)	96.4	50.9-100.0

The results from the full multiple linear regression models are available in Additional file 1. We present the results for the regression models containing the retained variables from the LASSO regression in the text and in Table 5. For productivity, older VHT age and VHT’s knowledge of danger signs were significantly associated with greater productivity at the *p* < 0.05 level; other VHT characteristics, such as level of engagement and competency, were not found to be significantly associated. VHTs who were 50 years or older had greater productivity than their counterparts younger than 50 years old (11.82, 95% CI: 4.03, 19.60), and for every ten-percentage point increase in knowledge of danger signs, the productivity score increased by 1.92 (95% CI: 0.01, 3.83). Years serving as a VHT had a marginally significant positive association with productivity (0.81, 95% CI: -0.05, 1.67) (Table 5). For performance, job satisfaction and knowledge of danger signs were significantly associated at the *p* < 0.05 level. For every unit increase in job satisfaction, the performance score increased by 1.46 (95% CI: 0.13, 2.80) and for every ten-percentage point increase in knowledge of danger signs, the performance score increased by 1.02 (95% CI: 0.05, 1.98). At the *p* < 0.10 level, mothers who were < 18 years old had VHTs whose performance was 5.62 units lower than mothers aged ≥18 (95% CI: -12.14, 0.90) and for every additional year of maternal education, the VHTs performed worse by 0.53 percentage points (95% CI: -1.08, 0.02) (Table 5).

**Table 5:** Covariates associated with productivity and performance, selected through LASSO regression

	Β	*p*
Productivity		
VHT age ≥ 50 (ref: < 50)	11.82 (4.03, 19.60)	0.003
Having access to motorcycle or bicycle	5.16 (−1.73, 12.06)	0.142
Knowledge of danger signs (expressed in percentage, unit of ten-percentage points)	1.92 (0.01, 3.83)	0.049
Years serving as VHT	0.81 (−0.05, 1.67)	0.066
Performance		
Job satisfaction score	1.46 (0.13, 2.80)	0.032
Knowledge of danger signs (expressed in percentage, unit of ten-percentage points)	1.02 (0.05, 1.98)	0.039
Years of mother’s schooling	−0.53 (−1.08, 0.02)	0.057
Mother aged < 18 (ref: ≥18)	−5.62 (−12.14, 0.90)	0.091
Number of child health issues raised by the mother	−1.43 (−3.28, 0.41)	0.128
Availability of flooring in VHT household	−2.58 (−6.28, 1.12)	0.171

## Discussion

CHVs serve a critical role in providing life-saving health interventions in low-resource settings, particularly among the hardest-to-reach populations. Productivity and performance are both key measures in assessing how effectively CHVs are serving their respective communities. We observed in our study that there is minimal correlation between measures of VHT productivity and performance in two sub-counties of Busia District, Uganda, and we only observed that knowledge of danger signs were significantly associated with both measures. This implies that while there was no evidence that performance was being sacrificed for increased productivity or vice versa, there was also no evidence to suggest that improvements in one would contribute to improvements in the other, or that there are specific characteristics of VHTs or their environments that could be targeted to improve both. This highlights the need for conscious policy planning that recognizes that these two components of VHT effectiveness may need potentially integrated, but nonetheless independent, interventions. Also, VHTs require ongoing practical training and supportive supervision to develop and maintain competencies in identifying danger signs in pregnant mothers and children. We believe our findings are generalizable to the greater VHT system in Uganda and to similar CHV models.

### Factors affecting productivity and performance

A desk review on health worker productivity highlighted knowledge and skills, motivation, and the work environment (workload, supportive supervision, supplies and equipment, respect) as determinants of productivity [5]. We were not able to find a statistical relationship between productivity and those characteristics that we were able to capture in our study. This may be attributable to several factors. One, contextual differences may affect how influential each factor is on productivity. Dieleman et al. reported that health resource management interventions on CHW performance have shown different effects in different contexts [22], and the same issue likely applies to productivity. We may not have successfully measured or captured the factors that influence productivity specifically among the CHVs in our context. Two, certain factors may be modifying the relationship of other factors to productivity. Due to our small sample size, we were unable to examine interactions between various independent factors. In the existing literature, a study comparing interventions that focused on CHW training noted that the selection process of the CHWs (i.e., by the beneficiary community themselves vs. by the political establishment) and the targeted need of the community (unmet vs. met need) were contextual factors that modified the intervention outcome [7]. Similar analyses on effect modification should be conducted in the future with productivity as an outcome, as much of the existing literature focuses on performance.

VHT characteristic of being older than 50 years of age (ref: 35- < 50 years of age) and knowledge of danger signs were statistically significantly associated with productivity. Independent from age, number of years of serving as a VHT was marginally significant. Engagement, incentives, recognition, availability of equipment, and supervision were not found to be associated with productivity in this study. While we were unable to identify existing studies examining the relationship between CHV age and productivity, other studies have shown mixed findings regarding health worker’s age and performance [23]. A study in India reported that absenteeism was highest among women aged 25-40 due to pregnancies and other maternal health issues [24]. Another study in Kenya, examining age groups ranging from a “below 30” category to an “above 60” category, saw highest performance among women aged 40-50 years [25]. The authors attributed this to social stability within that age group. It is also important to note that experience may not consistently correlate with improved performance or productivity; a study examining the performance of health workers on neonatal resuscitation in Malawi saw that health workers with longer years of experience showed the poorest levels of performance. That study hypothesized that newer standards of care may need reinforcement among individuals who have more experience [26]. The fact that older mothers may prompt better quality performance is consistent with other literature [27]. The additional years of maternal education was associated with poorer CHV performance, however the effect size is negligible and it is unclear what the operating mechanism would be.

The magnitude of effect sizes was quite small for performance, thus whilst some indicators were statistically significantly associated, they did not appear to have a meaningful bearing on performance. Job satisfaction was a factor significantly associated with performance, however not with productivity; previous studies have described the relationship with performance as well. Several studies from different contexts have highlighted low salary and lack of professional development opportunities as key components influencing job satisfaction [28–30]. The salary issue is notable, as financial incentives have been reported in certain situations to have a negative impact on motivation [22]. This distinction between salary and financial incentive may be reflective of CHWs valuing respect and formalized support from the government and the health system. In a separate study on VHT motivations, VHTs expressed a desire to receive a stipend that is approximately 75% of the minimum public sector wage and for the VHT role to become something that resembles a full-time job [31]. A third of the VHTs indicated that their work negatively affected household income. Many of them expressed willingness to take on more work than the current workload, but it is unclear if and when the cost-benefit ratio will shift to deter volunteering their time, as the government utilizes VHTs for additional tasks. Using several different methodologies to calculate opportunity cost of CHV time used toward their work, the authors of the study recommended a monthly stipend of 5-10 USD for CHVs to cover basic expenditures and receive a commensurable reward. The lowest salary level within the Ugandan public health sector was 54 USD per month at the time of the study [31].

The Ugandan MoH is moving toward a salaried CHW program. A national assessment of 2610 VHTs in Uganda conducted in 2015 found that across the country, forms of monetary and non-monetary motivation for VHTs have been irregular, varying across implementing partners and districts, undermining coordination and retention of VHTs. While the government’s VHT Operational Guidelines stipulate the various forms of motivation for VHTs, it does not explicitly state how these incentives should be equitably distributed and who should provide them. Qualitative data from VHT interviews overwhelmingly supported regular financial form of motivation. Such regular financial incentives were advocated for in the Ministry’s 2015 Community Health Extension Workers Strategy which recommends replacing the VHT program with full-time salaried community health extension workers who would be government employees [32]. The true impact of financial compensation on job performance, retention, and sustainability has not been properly examined in many contexts; Uganda’s transition may provide a unique opportunity to assess how compensation affects CHV or CHW programs.

One of the strengths of our study is that we were able to conduct direct observations to assess key productivity and performance measures. Many of the prior published studies of CHV performance have been done in health facilities or in situations that do not represent the context within which they provide their services. The observations allowed for the validation of danger sign assessment and for measurement of the length of a home visit, permitting quantification of productivity. There has been documented overestimation of performance during observations, or the “Hawthorne effect”, compared to situations where CHVs or CHWs were unaware that they were being observed. However, without direct observation, performance on danger sign assessment could not be compared and interviews would be subject to biased responses.

### Limitations

While our study focused on the performance and productivity of CHVs, guaranteeing high performance and high productivity alone does not necessarily lead to improved health outcomes. We only report the CHW-level side of health care access and do not address the end-user in our study, and as such were unable to determine the relationship between CHV productivity or performance and health outcomes, as that went beyond the scope of the study. Socioeconomic and cultural barriers to referral completion have been discussed extensively [33–35], and high quality of care at referral facilities is not guaranteed. For severe cases in which referral completion is vital to saving a life, the factors beyond the first point-of-contact with the CHV will need to be addressed before CHV programs can maximize their utility. There appeared to be variability in productivity levels, but we did not have sufficient information to understand the variation.

The cross-sectional study design does not allow us to determine directionality of the associations we observed here. For instance, we observed a negative association between supervisory visits and performance. This may be due to worse-performing VHTs being targeted for additional supervisory visits. Also, we were unable to collect complete data on the full set of tasks in the VHT basic package of services, thus we could not capture productivity and performance scores that reflect the entirety of a VHT’s responsibilities. This limitation may have led to an under-estimation of VHTs’ productivity. Finally, with our small sample size, the limited variability in the dependent and/or the independent variables may have prevented us from capturing some of the associations.

## Conclusion

We observed no correlation between productivity and performance among VHTs in Eastern Uganda. We also did not identify variables that were associated with both measures. We found that older VHT age and knowledge of danger signs were positively associated with productivity and that job satisfaction and knowledge of danger signs were positively associated with performance. The lack of correlation between productivity and performance in this study suggests that interventions to improve CHV effectiveness may affect each of these two dimensions of effectiveness differently. While our study did not suggest that productivity gains must necessarily be sacrificed for performance gains, CHV improvement interventions, particularly for counselling and support tasks associated with community-level services, may not affect both. CHW interventions include improved clarity in roles, competency development, and supportive supervision and feedback [8, 36]. We recommend that both dimensions of CHV effectiveness be monitored to evaluate the overall program impact of CHV improvement interventions. Consideration of the independent effects of improvement interventions on productivity and performance is likely to be relevant even as the Government of Uganda considers moving away from a CHV to a paid CHW model.

## Additional files

Additional file 1: Supplemental file. Contains Tables S1, S2, S3, S4 and Supplemental Text 1. (DOCX 71 kb)

Additional file 2: Data collection file. Contains data collection tools. (DOCX 153 kb)

## Abbreviations

CHV: Community health volunteer
CHW: Community health worker
CI: Confidence interval
CSI: Composite Services Index
DEFF: Design effect
HIV: Human immunodeficiency virus
PCA: Principal Component Analysis
USD: United States dollar
VHT: Village Health Team
