# Supplementary material for: Measuring productivity and its relationship to community health worker performance in Uganda: a cross-sectional study
Source: BMC Health Serv Res. 2018 May 9;18:340. doi: 10.1186/s12913-018-3131-9 (PMC5941461; doi:10.1186/s12913-018-3131-9)
Supplement: Supplementary file 1 — Supplemental file. Contains Tables S1, S2, S3, S4 and Supplemental Text 1. (DOCX 71 kb) [file 12913_2018_3131_MOESM1_ESM.docx]

**Table S1. Uganda Ministry of Health basic package of VHT services**

| **Tasks** | **Activities** |
| --- | --- |
| 1. Record and examine | 1. Make a map of the village 2. Fill in and maintain the village register 3. Fill in and submit Summary Report Form 4. Analyze the information in the village register |
| 1. Visiting village members* | 1. Observe health behaviour, practices, hygiene and health condition of household members 2. Share information and give advice about healthy habits to neighbours during home visits 3. Share information with village groups during health talks 4. Follow up with patients at home |
| 1. Helping save lives* | 1. Recognize danger signs 2. Refer people needing health care to health unit 3. Help ensure that all children are immunized 4. Counsel every pregnant women about timely antenatal care (ANC) visits, newborn care, and timely post-partum (after delivery) checks 5. Conduct post partum (after delivery) home visits to advise families about newborn and maternal care 6. Advise people on basic first aid for minor injuries and illnesses |
| 1. Link the village and the health unit | 1. Plan activities with the health unit staff 2. Support health unit activities in the village 3. Share and plan with the village leaders 4. Notify health unit staff immediately about “reportable” diseases 5. Encourage those who want information or help with child spacing to go to the health unit |
| 1. Mobilize the village | 1. Mobilize village members for health activities |
| 1. Hold monthly team meetings | 1. Hold monthly VHT team meetings |

*Focus of this paper.

Citation: Uganda Ministry of Health. Village Health Team – Participants Manual for Village Health Team Members. <http://ccmcentral.com/wp-content/uploads/2014/04/VHT-Participant-Manual-Uganda_MOH-Uganda_n.d..pdf>

**Table S2: Independent factors of interest, as identified through literature or through expert consultation**

| **Influencing factors** | **Description of independent variables** |
| --- | --- |
| 1. VHT | Demographics: Sex, age, marital status^ |
|  | Occupation (Kalyango et al. 2012) |
|  | Economic: Type of floor (eg - dirt/cement/carpet or tile), type of roofing and type of external walls at home^ |
|  | Length of time as a VHT^ |
|  | Reason for becoming a VHT (Taylor 2009) |
|  | How VHT was selected^ |
| 1. VHT engagement in their work and with other VHTs | Job satisfaction+   - Ratings on a 5 point likert-type scale on statements relating to being proud to be a VHT, feeling happy with the VHT work, ease of interaction with others, being known for their reliability as a VHT |
|  | Change agent+   - Ratings on a 5 point likert-type scale on statements relating to helping other VHTs to learn new skills, encouraging other VHTs to discuss challenges, applying new skills in their work, suggesting solutions, and giving feedback to other VHTs |
|  | Accountability+   - Ratings on a 5 point likert-type scale on statements relating to seeing their VHT work through to completion, completing tasks on time, perception of clarity of goals, evaluating their own performance |
| 1. VHT competencies | Background education (Rowe et al. 2007)   - Number of years of schooling completed |
|  | Initial VHT training^   - Completion of initial training - Time since initial training |
|  | In-service training (Kalyango et al. 2012; Naimoli et al. 2006; Zurovac et al. 2004)   - Completion of any other training while they have been volunteering as a VHT - Topic of training completed |
|  | Knowledge of danger signs in pregnant women, newborns and children |
|  | Perceived adequacy of competencies on a five point likert type scale+ |
| 1. Catchment area | Number of households served (Jaskiewicz and Tulenko 2012; Kalyango et al. 2012) |
|  | Distribution of households served (Jaskiewicz and Tulenko 2012; Kalyango et al. 2012) |
| 1. Incentives | Financial incentives received (Alam, Tasneem, and Oliveras 2012; Rowe et al. 2007) |
|  | Non-financial incentives received (R and L 2012; Rowe et al. 2007) |
| 1. Recognition | Perception of recognition by stakeholders- health workers, community, local government, government (Alam et al. 2012; Jaskiewicz and Tulenko 2012) |
| 1. Supervision | Frequency of supervision (Hadi 2003) |
|  | Who supervises the VHT (Taylor 2009) |
|  | Perceived adequacy of supervision against VHT program supervision requirements [5, 12] |
| 1. Linkages and stakeholder relationships | Distance from the village to the health facility^ |
|  | Perception of supportive relationships with health facilities, local government structures, community structures and clients (Alam et al. 2012; Jaskiewicz and Tulenko 2012; Kalyango et al. 2012) |
|  | Utilization of networks and relationships with community structures to provide VHT services^ |
|  | Frequency communication with health facilities (Kalyango et al. 2012) |
| 1. Equipment and supplies | Availability of basic inputs required for provision of VHT services. For example: soap, clean water. (Jaskiewicz and Tulenko 2012) |
|  | Availability of materials required for record keeping and referrals. For example: referral forms, registers. (Jaskiewicz and Tulenko 2012) |
|  | Ready access to means of free transportation for VHT work (Kalyango et al. 2012) |
|  | Availability of a cell phone |
|  | Availability of essential commodities (Jaskiewicz and Tulenko 2012; Kalyango et al. 2012; Stekelenburg, Kyanamina, and Wolffers 2003; Taylor 2009) |
|  | Perceived adequacy of essential commodities (Jaskiewicz and Tulenko 2012) |
|  | Use of job aids during interactions with clients (Rowe et al. 2007) |
| 1. Client factors | For the child case observed (Rowe et al. 2007)   - Age of child - Number of complaints described by caregiver during interactions |
|  | Mother counselling observed^   - Age of mother - Number of years of schooling completed by the mother |

*+Adapted from a study of health worker engagement in Tanzania* *(Kundy and Wuliji 2012)*

^*Recommended by the study stakeholder consultation group*

**Table S3: Principal Component Analysis component loadings for first component of selected groupings of variables**

| **Grouping of variables** | **Variance explained by first component** | **Composition of first component** |
| --- | --- | --- |
| Job satisfaction | 63.1% | I am proud to be VHT: 0.46  I feel happy with the VHT work that I do: 0.46  I can interact easily with other VHTs: 0.43  I am known by others I work with for my reliability in my VHT work: 0.49  I have the knowledge and skills to do my job well: 0.39 |
| Change agent | 55.8% | I help other VHTs in my village to learn new skills: 0.43  I encourage other VHTs in my village to discuss challenges: 0.52  When I learn new skills, I apply them in my VHT work: 0.47  I suggest solutions when discussing challenges with other VHTs in my village: 0.40  I give feedback to other VHTs in my village on their performance: 0.41 |
| Accountability | 53.0% | I stay on the job until I complete my tasks: 0.53  I complete my VHT tasks on time: 0.60  The goals of my VHT work are very clear to me: 0.52  I evaluate my own work performance: 0.31 |
| Perceived quality of supervision | 57.8% | Checked to see if I had enough medicines, supplies, and equipment to do my work: 0.71  Provided feedback on any medicines I gave to clients: 0.71  Helped me to find solution to problems: 5.3 |

**Table S4: Associations between independent factors and productivity / performance, full regression model**

|  | ***Productivity*** | | ***Performance*** | |
| --- | --- | --- | --- | --- |
|  | **β** | ***p*** | **β** | ***p*** |
| Sex (female, ref: male) | -2.94 (-10.57, 4.70) | 0.451 | 0.04 (-3.69, 3.77) | 0.982 |
| Young VHT age, <35 (ref: ≥35) | -0.90 (-8.94, 7.15) | 0.827 | -1.24 (-5.19, 2.71) | 0.538 |
| Older VHT age, ≥50 (ref: <50) | 8.60 (-0.48, 17.69) | 0.063 | -1.15 (-5.61, 3.32) | 0.615 |
| Has flooring at home | 0.74 (-7.03, 8.52) | 0.852 | -2.69 (-6.49, 1.12) | 0.167 |
| Education (years) | 0.23 (-1.37, 1.82) | 0.780 | -0.50 (-1.28, 0.29) | 0.212 |
| Number of years serving as a VHT | 0.81 (-0.14, 1.77) | 0.096 | 0.00 (-0.47, 0.47) | 0.989 |
| Job satisfaction score | 0.37 (-2.44, 3.19) | 0.796 | 1.64 (0.26, 3.02) | 0.020 |
| Change agent score | 0.69 (-2.22, 3.42) | 0.677 | 0.06 (-1.29, 1.41) | 0.931 |
| Accountability score | 1.44 (-1.65, 4.53) | 0.361 | -0.38 (-1.90, 1.14) | 0.624 |
| Knowledge of danger signs (per ten percentage point increase) | 1.68 (-4.16, 3.77) | 0.116 | 1.32 (0.30, 2.34) | 0.011 |
| Received financial incentive | 6.89 (-4.91, 18.69) | 0.252 | -0.12 (-5.91, 5.68) | 0.968 |
| Perceived health facility as supportive | 3.67 (-8.62, 15.96) | 0.558 | 0.81 (-5.23, 6.86) | 0.792 |
| Number of supervisory visits in the last 3 months | 1.33 (-1.84, 4.50) | 0.411 | -1.28 (-2.82, 0.26) | 0.104 |
| Supervisory content score | 0.77 (-4.58, 6.13) | 0.777 | -0.51 (-3.03, 2.01) | 0.691 |
| Number of supplies | -0.40 (-3.93, 3.13) | 0.825 | 0.26 (-1.46, 1.99) | 0.764 |
| Transport availability | 3.44 (-4.04, 10.93) | 0.367 | -2.97 (6.63. 0.68) | 0.111 |
| Cell phone availability | 0.04 (-8.33, 8.42) | 0.992 | 2.09 (-2.04, 6.21) | 0.321 |
| Mothers aged <18 (ref: ≥18) | -0.41 (-13.81, 12.99) | 0.952 | -6.68 (-13.26, -0.09) | 0.047 |
| Mothers aged >35 (ref: ≥35) | 1.73 (-0.76, 13.22) | 0.768 | -3.19 (-8.83, 2.46) | 0.268 |
| Years of mother’s schooling | -0.27 (-1.44, 0.89) | 0.648 | -0.66 (-1.24, -0.09) | 0.023 |
| Child’s age (≥1 month old, ref: <1 month) | 3.77 (-3.89, 11.43) | 0.335 | 0.62 (-3.11, 4.36) | 0.743 |
| Number of child health issues described by mother | 1.91 (-2.06, 5.88) | 0.346 | -1.46 (-3.39, 0.47) | 0.138 |

**Supplemental Text 1. Calculation of Composite Service Index**

The World Bank study accounted for different cadres of health workers by taking a weighted average of the mix of the workforce, with weights representing the salaries of each cadre (9). Weighted sums were not computed for VHTs in this adapted methodology as we assume that VHTs are homogenous.

$$VHT productivity= \frac{Total Composite Services Index \left( CSI \right)by a sample of VHTs in a district in 3 months}{Total number of VHTs sampled in the district}$$

The Composite Service Index (CSI) aggregates the health services included in the analysis (CSI). The CSI represents the sum of the Composite Activity Index (CAI) across the service categories. The CAI is the weighted volume of component tasks for a service category, recognizing that services included multiple tasks. Analysis in this study examined both the total aggregate productivity across the two categories (CSI per VHT) and for each service category (CAI per VHT). The CSI and CAI were expressed as service equivalents.

CSI = *a*X_1_ + *b*X_2_

- X_1 – 2_ are the volumes of outputs corresponding to the respective CAI
- *a, b* are weights (relative time use) assigned to respective volumes of outputs of the two service categories respectively
