# Supplementary material for: Measuring productivity and its relationship to community health worker performance in Uganda: a cross-sectional study
Source: BMC Health Serv Res. 2018 May 9;18:340. doi: 10.1186/s12913-018-3131-9 (PMC5941461; doi:10.1186/s12913-018-3131-9)
Supplement: Supplementary file 2 — Data collection file. Contains data collection tools. (DOCX 153 kb) [file 12913_2018_3131_MOESM2_ESM.docx]

**PERFORMANCE ASSESSMENT – RECORD REVIEW CHECKLIST**

**Background Information**

| **Sub-county code** | **_____________** |  | **VHT code** | **_______________** |
| --- | --- | --- | --- | --- |
| **Parish code** | **_____________** |  | **Data collector code** | **_______________** |
| **Village code** | **_____________** |  | **Study supervisor code** | **_______________** |
|  |  |  | **Date** | **_______________** |

**Informed consent checklist**

**Participant(s) given the study information sheet**

**Consent instruction sheet has been followed**

**Participant(s) verbally consented to participation**

I hereby sign to verify that I have completed the informed consent instructions: ___________________

**Instructions**

1. *Explain to the VHT that you would like to observe how they keep records.*
2. *Record your observations on this form as outlined below.*

_____________________________________________________________________________________

| **SECTION 1: KEEPING UP-TO-DATE RECORDS** | | | | | | | | | |  |  |
| --- | --- | --- | --- | --- | --- | --- | --- | --- | --- | --- | --- |
| **1.THE VILLAGE MAP** | | | | | | | | |  |  |  |
|  | | *Ask the VHT if you can look at his/her Village Map. Follow the instructions below.* | | | | | | | |  |  |
| ***1.1 Look for symbols 1.1.1 to 1.1.5 on the village map*** | | | | | | | | | |  |  |
| *If you see this symbol on the map, randomly select one.* | | | | | *Ask to go to the household and verify if, within a period of three months from the survey date the following are accurate* | | | | *Tick the appropriate box* |  |  |
|  | 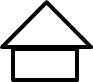1.1.1 | | | | The household has had NONE of the following: a pregnant mother, a child less than 5 years of age and no death has occurred | | | | Yes  No |  |  |
|  |  |  |  |  |  |  |  |  |  |  |  |
|  | 1.1.2 | | | 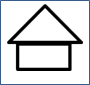 | A pregnant woman lives in the household | | | | **Yes  No** |  |  |
|  | 1.1.3 | | | 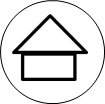 | A child less than 5 years of age lives in the household | | | | **Yes  No** |  |  |
|  | 1.1.4 | | | 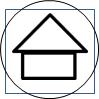 | A pregnant woman AND a child lives in the same household | | | | **Yes  No** |  |  |
|  | 1.1.5 | | | 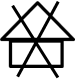 | A member of the household died within the last one month | | | | **Yes  No** |  |  |
|  | | | | | | | |  | |  |  |
|  | | | | | | | | | | |  |
| **1.2** | | | **Now *ask a village leader (e.g. LC 1) or elder to list the households to his knowledge that have experienced the following events in the past one month. Take note of the respective households which have experienced the events in question. Then ask the VHT to indicate where the household is marked on the map.*** | | | | | | *Tick the appropriate box* |  |  |
| *After the elder has listed the following* | | | | | | *Refer to the map and check if all the listed events are marked on the map with the appropriate symbols* | | | Yes  No |  |  |
| 1.2.1 | | | Households with a pregnant mother in the past one month | | | Are all listed households with a pregnant mother marked on the village map with this symbol? | 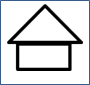 | | Yes  No |  |  |
| 1.2.2 | | | Households with a child less than 5 years of age in the past one month | | | Are all listed households with a child less than 5 years marked on the village map with this symbol? | 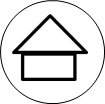 | | Yes  No |  |  |
| 1.2.3 | | | Households with both a pregnant mother and a child less than 5 years of age in the past one month | | | Are all listed households with both a pregnant mother and a child less than 5 years of age marked on the village map with this symbol? | 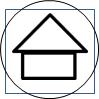 | | Yes  No |  |  |
| 1.2.4 | | | Households where a member has died in the past one month | | | Are all listed households where a member has died marked on the village map with this symbol? | 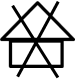 | | Yes  No |  |  |

1.2.5 *Ask the VHT to explain how they usually gather information to update the village map:*

<Tick all responses mentioned>

<Keep probing: *Any other?>*

1. Going physically from house to house

2. Through networks (eg – friends, relatives, church, school, other groups in the community)

3. Through community members that come to the VHT to directly share any updates with them

4. Other (*describe*): _________________________________________________________________

_____________________________________________________________________________________

**2.0 THE VILLAGE REGISTER**

*Explain to the VHT that you would like to see the village register. Follow the instructions below.*

| **2.1** | ***Retrieve the village register. Note the last reporting month and the number of households registered on the reporting date.*** | *Circle the correct option* | | |
| --- | --- | --- | --- | --- |
|  |  |  |  |  |
| a) | The last reporting date is within 90 days from today (day of survey); or within Apr – Jun 2013 | Yes | No |  |
| b) | Number of households registered in the last reporting period (Apr – Jun 2013) |  | | |
| **2.2** | ***Now, randomly select one of the household numbers. Visit the selected household, carrying the register along. For the selected village verify the accuracy of the following data included on the register with the head(s) of the household.*** |  | | |
|  | Write the number of the seledted household in the space provided |  | | |
|  |  |  | | |
| **2.3** | **The register correctly contains the following data about household members for the last reporting date**  ***From (D) to (O), check NA if the event did not occur during the reporting period*** | *Tick one for each row* | | |
| A | Names of all members | Yes | No |  |
| B | Sex of all members | Yes | No |  |
| C | Age of all household members | Yes | No |  |
| D | All deaths | Yes | No | NA |
| E | Children > 5 years who are not in school are indicated | Yes | No | NA |
| F | Number of adolescent and adults on family planning methods | Yes | No | NA |
| G | Adults on ART | Yes | No | NA |
|  | *If there was a pregnant woman by the reporting date* |  |  |  |
| H | Delivery month | Yes | No | NA |
| I | Number of antenatal care (ANC) visits to at health facilities | Yes | No | NA |
| J | History of danger signs | Yes | No | NA |
|  | *Concerning only children 5 years and below (If the household does not contain any child under 5 years of age, check NA)* |  |  |  |
| K | Records on immunisation | Yes | No | NA |
| L | Records of de-worming | Yes | No | NA |
| M | Records of Vitamin A | Yes | No | NA |
| N | Growth monitoring records (yellow MUAC, red MUAC) | Yes | No | NA |
| O | Use of Insecticide Treated Nets s | Yes | No | NA |
|  |  |  |  |  |
| **2.4** | **Move around the home to verify if all the checked items are available in the home; or if an item that was available during Apr – Jun 2013 has not been recorded in the register .**  **The register correctly documents the presence of the following in the household** |  | | |
| A | Protected or safe water source | Yes | No |  |
| B | Safe drinking water | Yes | No |  |
| C | Kitchen | Yes | No |  |
| D | Drying rack for dishes | Yes | No |  |
| E | Rubbish pit | Yes | No |  |
| F | Latrine | Yes | No |  |
| G | Hand-washing with soap near latrine | Yes | No |  |
| **2.5** | **Now turn to the section of the Village Register which summarises the data in the previous sections of the register.** |  | | |
|  | A summary report was compiled for the most recent quarter | Yes | No |  |

2.5 *Ask the VHT to explain how they usually gather information to update the village register:*

1. Going physically from house to house

2. Through networks (eg – friends, relatives, church, school, other groups in the community)

3. Through community members that come to the VHT to directly share any updates with them

4. Other (*describe*): _________________________________________________________________

_____________________________________________________________________________________

**CHECKLIST FOR OBSERVING A VHT DURING A HOME VISIT**

**Background Information**

| **Sub-county code** |  |  | **VHT code** |  |
| --- | --- | --- | --- | --- |
| **Parish code** |  |  | **Data collector code** |  |
| **Village code** |  |  | **Study supervisor code** |  |
| **Household number** |  |  | **Date** |  |
|  |  |  |  |  |

**Informed consent checklist (administered to head of household)**

**Participant(s) given the study information sheet**

**Consent instruction sheet has been followed**

**Participant(s) verbally consented to participation**

I hereby sign to verify that I have completed the informed consent instructions: ___________________

**Instructions**

1. *Explain to the VHT that you would like to observe how a routine home visit is carried out.*
2. *Ask the VHT if you can join him/her on their next home visit and agree on a date and time for the visit. Take the form of transportation that the VHT would normally take.*
3. *Ask the VHT to introduce you to the head of the household. Introduce yourself to the head of the household and explain the purpose of the study briefly, explain the purpose of your visit, and that participation is voluntary. Request their consent. If consent is given, sign above to document that verbal consent was given.*
4. *Observe the process un-obtrusively and record your observations on this form as outlined below.*

| 1. **Transportation used** | |
| --- | --- |
| - 1. To the home visit (describe) |  |
| - 1. Back from home visit (describe) |  |
| 1. **Times** | |
| - 1. Time of departure from the VHT’s home | **____:____** (hr. /min) AM / PM |
| - 1. Time of arrival at the selected household: | **____: ____** (hr. /min) AM / PM |
| - 1. Time of departure from the selected household | **____: ____** (hr. /min) AM / PM |
| - 1. Time of arrival at the VHT’s home | **____: ____** (hr. /min) AM / PM |
| 1. **Home visit** | |
| - 1. VHT explains purposes of visits/problems to be discussed* | Yes No |
| - 1. VHT uses a job aid during the home visit | Yes No |
| - 1. VHT addresses the family politely and respectfully | Yes No |
| - 1. VHT explains solutions to any identified problems | Yes  No  NA (household does not have any problems) |
| - 1. VHT provides relevant advice about health habits | Yes No |
| - 1. VHT facilitates an agreement with the family on what actions should be taken | Yes No |
| - 1. VHT facilitates an agreement with the family on who will do what | Yes No |
| - 1. VHT facilitates an agreement with the family on when actions will be taken by | Yes No |
| - 1. VHT plans a follow-up visit to check if changes are made | Yes No |
| - 1. VHT makes a post-visit note after departing the household visited** | Yes No |

** Note that the purpose of the visit may be implied in the initial informal interaction/conversation between the VHT and the household head, rather than being explained formally*

***Note: if this does not happen while you are still with the VHT, follow up the next day to find out if the VHT made a note about the visit before you depart. If he answers in the affirmative, ask to see the note.*

- 1. ***Ask the VHT how they usually identify which households need a home visit*:**

<tick all that apply>

1. Going physically from house to house

2. Through networks (eg – friends, relatives, church, school, other groups in the community)

3. Through community members that come to the VHT to directly share any updates with them

4. Other (*describe*): _____________________________________________________________

__________________________________________________________________________________

**CHECKLIST FOR ASSESING DANGER SIGNS IN A PREGNANT MOTHER**

**Background Information**

| **Sub-county code** |  |  | **VHT code** |  |
| --- | --- | --- | --- | --- |
| **Parish code** |  |  | **Data collector code** |  |
| **Village code** |  |  | **Study supervisor code** |  |
|  |  |  | **Date** |  |

**Informed consent checklist**

**Participant given the study information sheet**

**Consent instruction sheet has been followed**

**Participant verbally consented to participation**

I hereby sign to verify that I have completed the informed consent instructions: ___________________

**Instructions:**

1. *Explain to the VHT that you would like to observe how an assessment of a pregnant mother is carried out.*
2. *Ask the VHT if you can join him/her on their next assessment of a pregnant mother and agree on a date and time for the visit/observation of self-reporting pregnant mother.*
3. *If travelling on a home visit, take the form of transportation that the VHT would normally take.*
4. *Ask the VHT to introduce you to the head of the household. Introduce yourself to the head of the household and explain the purpose of the study briefly, explain the purpose of your visit, and that participation is voluntary. Request their consent. If consent is given, sign above to document that verbal consent was given.*
5. *Request the VHT to carry out a typical assessment on the mother. Observe the process un-obtrusively and record your observations on this form as outlined below.*
6. *After the VHT’s assessment, conduct a gold standard assessment and document your observations in the form. Be sure that this is not done in the VHT’s presence.*
7. *Thank the VHT and family for allowing you to observe the visit*

****Refer to the list of likely medical emergencies in a pregnant mother. If you can see that there is an urgent medical emergency stop the observation immediately. Ask the VHT and head of household to take immediate action for the pregnant mother to be transported to the nearest health facility.**

**Tick here if an urgent medical emergency was observed**

**Tick here if the urgent medical emergency was followed up with immediate action**

**CHECKLIST FOR ASSESING DANGER SIGNS IN A PREGNANT MOTHER**

In 1 to 4 below, tick *NA if mother comes to VHT*

1. Record the time you leave the VHT’s home: **____: ____** (hr. /min) AM / PM;  NA
2. Record the exact time that the consultation begins: **____: ____** (hr. /min) AM / PM;  NA
3. Record the exact time that the consultation ends: **____: ____** (hr. /min) AM / PM;  NA
4. Record the exact time that you arrive back at the VHT’s home:**____: ____** (hr. /min) AM / PM; NA

| **Danger signs** | **Pregnant mother** | **VHT** | | **Gold standard assessor (GSA)** |
| --- | --- | --- | --- | --- |
|  | Information volunteered | VHT asks | VHT finds symptom present | GSA finds symptom present |
| *Waberengehowo ohudachirira nga hubitira mu buhasi*   1. History of vaginal bleeding | Yes  No | Yes  No | Yes  No | Yes  No |
| *Wabalireho omubiri mumasawa24*   1. Fever or history of fever in the past 24hours | Yes  No | Yes  No | Yes  No | Yes  No |
| *Omutwe ohuhomaka namani nende emoni obutabona bulayi mungeri y’amabuliesi*   1. Severe headaches and blurred vision | Yes  No | Yes  No | Yes  No | Yes  No |
| *Omwana hasali okenda bilayi munda*   1. The baby has stopped moving | Yes  No | Yes  No | Yes  No | Yes  No |
| *Obechangaho omujoongu ohusingira mu buliri esiha sindi*   1. She is too weak to get out of bed | Yes  No | Yes  No | Yes  No | Yes  No |
| *Owulirangaho amachuni mulwena*   1. Severe pain in the lower part of the belly | Yes  No | Yes  No | Yes  No | Yes  No |
| Ohufimba hw’enjala, mumoni nende amakulu   1. Swellings of fingers, face, and legs | Yes  No | Yes  No | Yes  No | Yes  No |
| *Endumwa yadihe ebiha nibitola*   1. Water has broken before the expected time | Yes  No | Yes  No | Yes  No | Yes  No |
| *Obangalalangaho?*   1. History of convulsion | Yes  No | Yes  No | Yes  No | Yes  No |

* *For the following questions, tick ‘Yes’ if client volunteers history of complaint (without VHT asking) or answers “Yes” to VHT’s questions*

1. VHT uses a job aid during the consultation  Yes  No
2. VHT advises that pregnant mother needs to be referred to the health facility (write NA if VHT does not refer mother)  Yes  No  NA
3. VHT completed referral note  Yes  No  NA

***If NO but one is required, the gold standard assessor should write a referral note*

*Tick here if gold standard assessor wrote a referral note*

1. VHT reminds pregnant mother to go for her next antenatal care visit (ANC)  Yes  No
2. Ask the pregnant mother for her age: ___________ years

*Teba owesihaye ali asito emyaka kyaye ___________ emyaka*

1. Ask the pregnant mother how many years of school she has completed: _____________ years

*Teba owesihaye ali asito emyaka chiyamala mu lyekeero ________________ emyaka*

1. Ask the VHT to explain how they usually gather information on which pregnant mothers need to be assessed for danger signs <tick all that apply>:

Teba owa VHT akaniayeho koti lubateberesanga embosi echidira hu abesihaye bali asito hubanyale okonyeresaho hubudinyu bwa hechera mumera yohuba asito

1. Going physically from house to house

Ohukenda edaala hu daala

2. Through networks (eg – friends, relatives, church, school, other groups in the community)

Ohubitira muboholegana (koti – ebecha, abeho, abesayiro nende abebidiyiha bandi yomenyere

3. Through community members that come to the VHT to directly to see them

Ohubitira mubamenyi nomba abataha abecha okeniyaho VHT

4. Other (*describe*): Nebindi (bitotole) _____________________________________________________________________________________

**CHECKLIST FOR ASSESING DANGER SIGNS IN A SICK CHILD**

**Background Information**

| **Sub-county code** |  |  | **VHT code** |  |
| --- | --- | --- | --- | --- |
| **Parish code** |  |  | **Data collector code** |  |
| **Village code** |  |  | **Study supervisor code** |  |
|  |  |  | **Date** |  |

**Informed consent checklist**

**Participant given the study information sheet**

**Consent instruction sheet has been followed**

**Participant verbally consented to participation**

I hereby sign to verify that I have completed the informed consent instructions: ___________________

**Instructions**

1. *Explain to the VHT that you would like to observe how an assessment of a sick child aged 1 month to 5 years is carried out.*
2. *Ask the VHT if you can join him/her on their next assessment of a sick child and agree on a date and time for the visit/observation of self-reporting sick child.*
3. *If travelling on a home visit, take the form of transportation that the VHT would normally take.*
4. *Ask the VHT to introduce you to the head of the household. Introduce yourself to the head of the household and explain the purpose of the study briefly, explain the purpose of your visit, and that participation is voluntary. Request their consent. If consent is given, sign above to document that verbal consent was given.*
5. *Request the VHT to carry out a typical assessment on the child. Observe the process un-obtrusively and record your observations on this form as outlined below.*
6. *Following the VHT’s assessment, conduct a gold standard assessment and document your observations in the form. Be sure that this is not done in the VHT’s presence.*
7. *Thank the VHT and family for allowing you to observe the visit*

**** Refer to the list of likely medical emergencies in a sick child. If you can see that there is an urgent medical emergency stop the observation immediately. Ask the VHT and head of household to take immediate action for the sick child to be transported to the nearest health facility.**

**Tick here if an urgent medical emergency was observed**

**Tick here if the urgent medical emergency was followed up with immediate action**

**CHECKLIST FOR ASSESING DANGER SIGNS IN A SICK CHILD 1 – 59 MONTHS**

1. Record the time you leave the VHT’s home: **____: ____** (hr. /min) AM / PM
2. Record the exact time that the consultation begins: **____: ____** (hr. /min) AM / PM
3. Record the exact time that the consultation ends: **____: ____** (hr. /min) AM / PM
4. Record the exact time that you arrive back at the VHT’s home: **____: ____** (hr. /min) AM / PM
5. VHT asks about age of child  Yes  No
   1. *Confirm and note down the age of the child* ______ years and ______ months

***In the following questions,* c*heck ‘Yes’ if client volunteers history of complaint or answers “Yes” to VHT’s questions***

| Danger sign  **Obudinyu (esy’ekesa) esyekesa obudinyu bwahabi** | **Caregiver** | **VHT** | | | | **Gold standard assessor** | |
| --- | --- | --- | --- | --- | --- | --- | --- |
|  | Information volunteered (without VHT asking) | VHT asks | VHT finds symptom present | *VHT asks duration if symptom present | VHT records duration | Gold standard assessor finds symptom present | Symptom duration if present (days) |
| omubiri ohubala   1. Fever | Yes  No | Yes  No | Yes  No | Yes  No | Yes  No | Yes  No |  |
| *Omunyalalo*   1. Diarrhoea | Yes  No | Yes  No | Yes  No | Yes  No | Yes  No | Yes  No |  |
| *Nomba obutayera bilayi*   1. Cough or difficulty breathing | Yes  No | Yes  No | Yes  No | Yes  No | Yes  No | Yes  No |  |
| *Obutanyala ohungwa nomba ohununa*   1. Unable to drink or breast-feed | Yes  No | Yes  No | Yes  No | Yes  No |  | Yes  No |  |
| *Ohusala buli sy’olya*   1. Vomiting everything | Yes  No | Yes  No | Yes  No | Yes  No |  | Yes  No |  |
| *Sowamu endoolo, obujongu nende olumbuchu*   1. Abnormally sleepy, weak, or unconscious | Yes  No | Yes  No | Yes  No | Yes  No |  | Yes  No |  |
| *Ohubangalala*   1. Convulsions | Yes  No | Yes  No | Yes  No | Yes  No |  | Yes  No |  |

1. VHT uses a job aid during the consultation  Yes  No
2. VHT advises that child needs to be referred to the health facility  Yes  No
3. VHT completed referral note  Yes  No

***If NO but one is required, the gold standard assessor should write a referral note*

*Tick here if gold standard assessor wrote a referral note*

1. *Ask the VHT to explain how they usually gather information on which pregnant mothers need to be assessed for danger signs:*

*<tick all thatapply>*

1. Going physically from house to house

*Ohukenda edaala hu daala*

2. Through networks (eg – friends, relatives, church, school, other groups in the community)

*Ohubitira muboholegana (koti – ebecha, abeho, abesayiro nende abebidiyiha bandi yomenyere*

3. Through community members that come to the VHT to directly to see them

*Ohubitira mubamenyi nomba abataha abecha okeniyaho VHT*

4. Other (*describe*):

*Nebindi (bitotole):*

_____________________________________________________________________________________

**INTERVIEW WITH HEALTH FACILITY STAFF: HEALTH ASSISTANT (VHT SUPERVISOR)**

**(PERFORMANCE: LINKING THE VILLAGE WITH HEALTH FACILITIES)**

**Background Information**

| **Sub-county code** | **­­­** |  | **VHT code** |  | |
| --- | --- | --- | --- | --- | --- |
| **Parish code** |  |  | **Data collector code** |  | |
| **VHT’s village code** |  |  | **Study supervisor code** |  | |
|  |  | **Date** | |  | |
| **Health facility code** |  |  | | **Tick if respondent is the VHT’s direct supervisor** |  |
| **Title of the health worker** |  |  | |  |  |

**Informed consent checklist**

**Participant given the study information sheet**

**Consent instruction sheet has been followed**

**Participant verbally consented to participation**

I hereby sign to verify that I have completed the informed consent instructions: **___________________**

**Instruction**

1. *Arrange to go to the HF to which all the VHTs in the selected village are attached. If possible, arrange, with the help of your supervisor to have one interview day regarding the performance of all the VHTs attached to a particular HF*
2. *Carry a questionnaire per VHT linked to the HF*
3. *Follow the instructions on the consent form first, request verbal consent, document verbal consent if given and proceed*
4. *Read the following:* In particular, I would like to understand how a few selected VHTs who are attached to this health facility have been doing their work. These VHTs have been selected randomly. Your health facility has been selected because the sampled VHTs are attached to this health facility.
5. *Continue to read the questions below:*

I would suggest that we discuss each VHT, one at a time (**one questionnaire per VHT)**

1. Please tell me if, over the past 3 months, you have ever worked together with <<name of VHT>> from <<name of village>> (*circle one*)
   - - 1. Yes 2. No 97. Not sure

*If “Yes”, continue with interview; if “No” or “Not sure”, end the interview*

1. If yes, please describe the issues you have worked on together with <<name of VHT>> in the last 1 month (*let the in-charge mention the issues spontaneously, and quietly tick the following list once mentioned*)
   1. Planning for activities
   2. Supporting health facility activities in the village
   3. Notifying health facility staff about reportable diseases
   4. Referred patients to health facilities
   5. Other - (specify1): ___________________________
   6. Other - (specify2): ___________________________
   7. Other - (specify3): ___________________________
2. Overall how satisfied do you feel about the contribution of <<name of VHT>> to the health of those in the village? On a scale from 1 to 5, with 1 being very satisfied, and 2 being very dissatisfied:

1. Very satisfied  2.  3.  4.  5. Very dissatisfied

3a. Please explain why you chose the score that you gave (*write down as much detail as you can)*:

__________________________________________________________________________________

__________________________________________________________________________________

1. Regarding each of the following activities you have most recently worked on together with <<name of VHT>>, what is your view of how well they performed in each of these activities? On a scale from 1 to 5, with 1 being poor performance and 5 being excellent performance, what rating would you give?

| **Listed activities** | **Performance rating (1= poor, 5 = excellent)** |
| --- | --- |
| - 1. Planning for activities |  |
| - 1. Supporting health facility activities in the village |  |
| - 1. Notifying health facility staff about reportable diseases |  |
| - 1. Referred patients to health facilities |  |
| - 1. Other - (specify1): |  |
| - 1. Other - (specify2): |  |
| - 1. Other - (specify3): |  |

1. If <<name of VHT>> has referred any patients/clients to the health facility in the past 3 months, were any of the referrals accompanied by referral forms? (*circle one*)

1.Yes 2. No 97. Not sure

5a. If YES, are VHT referral forms kept at the health facility? (*circle one*)

1.Yes 2.No 97.Not sure

- - 1. *If YES to Q5, ask to see the referral form(s). Review it (them) and record the following for the last 3 months*
       1. Number of referral forms completed by VHT ___________
       2. Number of forms from VHT showing reasons for referral ______________
       3. Number of forms from VHT describing the pre-referral actions taken _____________

Thank you for your time

**OBSERVATION CHECKLIST – MONTHLY VHT MEETINGS**

**Background Information**

| ***Sub-county code*** |  |  | ***Data collector code*** |  |
| --- | --- | --- | --- | --- |
| ***Parish code*** |  |  | ***Study supervisor code*** |  |
| ***Village code*** |  |  | ***Date*** |  |
|  |  |  | ***Meeting participants (list):*** | ***VHT code:*** |
|  |  |  |  | ***VHT code:*** |
|  |  |  |  | ***VHT code:*** |
|  |  |  |  | ***VHT code:*** |
|  |  |  |  | ***Others (type):*** |
|  |  |  |  | ***Others (type):*** |

**Informed consent checklist**

**Participant(s) given the study information sheet**

**Consent instruction sheet has been followed**

**Participant(s) verbally consented to participation**

I hereby sign to verify that I have completed the informed consent instructions: ___________________

**Instructions**

1. *Explain to the VHT that you would like to observe a monthly VHT team meeting.*
2. *Agree on a date and time for the meeting. Take the form of transportation that the VHT would normally take.*
3. *Ask the VHT to introduce you to the organizer of the meeting. Introduce yourself to the organizer of the meeting and other meeting participants. Introduce the purpose of the study briefly, explain the purpose of your visit, and that participation is voluntary. Request their consent. If consent is given, sign above to document that verbal consent was given.*
4. *Observe the process un-obtrusively and record your observations on this form as outlined below.*
5. *At the end of the meeting, randomly select one of the VHTs and ask him/her to explain to you whether, and how, s/he has found the meeting useful.*
6. *Thank the organizers and the VHTs for allowing you to observe their meeting*

***General information***

| 1. ***Meeting organizer*** | |
| --- | --- |
| - 1. *Who organized the meeting?* | *VHT team leader*  *VHT*  *Other (describe):* |
| 1. ***Transportation used*** | |
| - 1. *To the meeting (describe)* |  |
| - 1. *Back from meeting (describe)* |  |
| 1. ***Times*** | |
| - 1. *Time of departure from the VHT’s home* | *____:____ (hr. /min) AM / PM* |
| - 1. *Time of arrival at the selected meeting place* | *____: ____ (hr. /min) AM / PM* |
| - 1. *Time of departure from the selected meeting place* | *____: ____ (hr. /min) AM / PM* |
| - 1. *Time of arrival at the VHT’s home* | *____: ____ (hr. /min) AM / PM* |
| 1. ***VHT Team meeting*** | |
| - 1. *The team leader or the VHT running the meeting explains purpose of the meeting* | *Yes No* |
| - 1. *All VHTs has had a chance to describe to the others the activities completed in the past one month and/or the challenges faced in implementing the activities* | *Yes No* |
| - 1. *Others react to the presentations made by each VHT and/or advises a VHT with a specific challenge on how to solve challenges met* | *Yes No* |
| - 1. *Did all VHTs in the village participate in this meeting?* | *Yes No* |
| 1. ***Discussion of data from VHT registers during team meeting (tick if yes)*** | |
| - 1. *VHTs discuss data from VHT registers during the meeting* | *Yes No* |
| - 1. *VHTs Identify all the bad and good health activities in the register and/or areas of concern* | *Yes No* |
| - 1. *VHTs add up the total number of people or households in the area of concern* | *Yes No* |
| - 1. *VHTs evaluate the implications of the data with regard to the behaviour of the people in their village* | *Yes No* |
| - 1. *VHTs draw conclusions about the behaviour of people in the village* | *Yes No* |
| - 1. *VHTs decide on the next steps to be done* | *Yes No* |
| - 1. *VHTs compile a monthly summary report during the meeting* | *Yes No* |

**After the monthly VHT team meeting**

*Discuss with one randomly selected VHT member for an interview. Ask the selected VHT to explain how useful the meeting has been.*

6. Enjihala y’oluhungano yayibonamu amahulundu?

Do you find the meeting useful?  Yes  No

7. Hulwasina eeh nomba haba?

Why or why not?

#

# VHT interview

**Background Information**

| **Sub-county code** |  |  | **VHT code** |  |
| --- | --- | --- | --- | --- |
| **Parish code** |  |  | **Data collector code** |  |
| **Village code** |  |  | **Study supervisor code** |  |
|  |  |  | **Date** |  |

**Instructions**

*Follow the instructions on the consent form first, request verbal consent, document verbal consent if given and proceed to read out the survey questions and document their responses on this form.*

**Informed consent checklist**

**Participant given the study information sheet**

**Consent instruction sheet has been followed**

**Participant verbally consented to participation**

I hereby sign to verify that I have completed the informed consent instructions: **___________________**

**ESITUNDU SYANJA (1)**

**SECTION 1**

**Sino situndu sy’ewunja hubihudiraho**

**This section collects background information about you.**

Enjibulwa y’obulonge (fundiha ndala)

1. Sex: (*circle one*)

Wesisacha Wesihasi

1. Male 2. Female

Mwosi siina n’omwaka kuwanjiraho ohuba VHT?s

1. In which month and year did you become a VHT?

_________ (omwosi)/_________ (omwaka)

______ (month) / _______ (year)

Bahudoola batye ohuba VHT? (fundihiraho hubino)

1. How were you selected as a VHT? (*circle all that apply*)

Nende abataha/ abamenyi hulukongo NOMBA Simanyire Ohayire obulirira

1. By my community OR 97. Not sure 98. Refused to answer

Nende esidiiha sya nasietuuse

2. By an NGO/donor funded project

Ebindi (totolaho mubusate)

3. Other (*write explanation*): ________________________________

Lwasina wahengawo ohuba VHT? Kesaho ohutula esonga chino mubulambase ohutula hu ehulundu chihirawo ohucha huchitari namani

1. Why did you decide to become a VHT? Please put these reasons in the order from most important to least important reasons.

*Beresa VHT olukalala lw’esonga chino obaberese bachitule mubulambase ohutula hu ehulundu nocha huchitari ehulundu muno. Sata ohwema nende olukala engeri yilurimo (1 = hulundu muno, 6 = sihulundu*

*Give printed reason cards to VHT with and ask them to put them into an order on the ranking card of most important to least important. Write the placing number the cards are put in (1= most important, 6 = least important).*

Ohoonya esitundu syange ____________

A. To serve my community _____

Ohweka ebindu biyaha ___________

B. To learn new things _____

Ohwongeraho emikisa change ch’ohunyola erubia _________

C. To improve my chances of earning money _____

Ohufuna ohusicha n’ohupahia ___________

D. To get recognition and respect ______

Hulw’obwekombi bwange hubidira huby’obusiroonyo ________

E. Because of my interest in health _____

Lwahuba abenango wange bandiririraho ___________

F. Because my family supported me _____

Oli nende emyaka kinga eky’obuhulundu?

1. How old are you?

____________ (emyaka) Simanyire NOMBA Ahayire ohukaluhamo

_________(years) 97 Not sure OR 98. Refused to answer

Oli nende abana banga?

1. How many children do you have?

_____ (abana) Simanyire NOMBA Ahayire ohukaluhamo

______ (children) 97 Not sure OR 98. Refused to answer

Oli omufumbo nomba oli mubudya

1. Are you married or currently in a long-term relationship? (circle one)

Eeh Haba Simanyire NOMBA Ahayire ohukaluhamo

1. Yes 2. No 97 Not sure OR 98. Refused to answer

Oli need obweru siina munyumba yawo?

1. What kind of floor do you have at home? (*circle one*)

Obwohuwoma

1. Not covered with either cement, tiles, concrete or carpet

Obusimitinge

2. Concrete

Obunyirise

3. Tile and/or carpet

Sihahasa

97. Not sure

Ahayire ohukaluhamo

98. Refused to answer

**ESITUNDU SY’OHUBIRI (2)**

**SECTION 2**

**Esitundu sino sy’otebaho hu bidira nende ebindu by’ohosesa biwebalahomo. Njicha ohuba ngahuteba onjekeseho bilala hubino**

**This section collects information about the equipment and supplies that you have. I will be asking you to show me some equipment and supplies.**

Onyala wahosesaho bilala hubino ohukendesa emirimo chawo nga sibahusasule nga VHT? (fundiihira ndala hu bino)

1. Can you at any time use any of these for transportation for your work without any payment as a VHT? (*circle all that apply*)

Engalangatani (fundihira ndala)

- 1. Bicycle (*circle one)*

Eeh Haba Sihahasa Ahayire okaluhamo

1 .Yes 2. No 97. Not sure 98. Refused to answer

Embiribindi (fundihira ndala)

- 1. Motorcycle (*circle one)*

Eeh Haba Sihahasa Ahayire okaluhamo

1 .Yes 2. No 97. Not sure 98. Refused to answer

Mugobe/omudingoyano (fundihira ndala)

- 1. Minibus (*circle one)*

Eeh Haba Sihahasa Ahayire okaluhamo

1 .Yes 2. No 97. Not sure 98. Refused to answer

Ebindi (totolaho)

- 1. Other (describe): ________________________________________

Onyala wanjekesaho noli nende muweweti (fundihira ndala)

1. Can you show me if you have a cell phone? (*circle one)*

Eeh (yekesa) Haba Ahayire okaluhamo

1. Yes (*was shown it)* 2. No 98. Refused to answer

Onyala wanjekesaho noli hoho nende omulabyo mungo wawo (fundihira ndala)

1. Can you show me if you have any soap at home? (*circle one*)

Eeh (yekesa) Haba Ahayire okaluhamo

1. Yes (*was shown it*) 2. No 98. Refused to answer

Onyala wanjekesaho nikali mbwe oli nende amachi malayi mayonjo? (fundihira ndala)

1. Can you show me if you have clean water at home? (*circle one)*

Eeh (yekesa) Haba Ahayire okaluhamo

1. Yes (*was shown it*) 2. No 98. Refused to answer

Onyala wanjekesaho esipima obubafu n’obunyifu bw’omubiri

1. Can you show me your thermometer? (*circle one*)

Eeh (yekesa) Haba Ahayire okaluhamo

1. Yes (*was shown it)* 2. No 98. Refused to answer

Onyala wanjekesaho epapulo chibahusindihirangoho munderero? (fundihira ndala)

1. Can you show me any blank referral forms that you have? (*circle one)*

Eeh (yekesa) Haba Ahayire okaluhamo

1. Yes (*was shown it)* 2. No 98. Refused to answer

Onyala wanjekesaho olukalala lwawo lwa VHT? (fundihira ndala)

1. Can you show me your VHT register? (*circle one)*

Eeh (yekesa) Haba Ahayire okaluhamo

1. Yes (*was shown it)* 2. No 98. Refused to answer

Obasa oti oli nende amalesi nende ebihuhonyereraho ebimala boosi abamadala kohonyererangaho? (fundihira ndala)

1. Do feel that you have enough medicines and supplies to meet the needs of the households you serve? (*circle one*)

Funangaho Ebiha bindi Sitera hufuna NOMBA Ahayire okaluhamo

1. Usually or always 2. Some of the time 3. Rarely or never OR 98. Refused to answer

**ESITUNDU SY’OHUDATU (3)**

**SECTION 3**

**Esitundu sino sibusa ebidira hu ndabirira ehola yemirimo**

**This section collects information about supervision**

Oholegananga nende enderero sina?

1. Which health facility are you attached to?

Elita ly’enderero _______________________________________________________

Health facility name: _________________________________________________________________

Mahabi kanga mumiosi chidatu chibitire kosyalireho oba wehasaho oluhiho ludira hu ndabirira?

1. How many times in the last three months have you had a visit or meeting for supervision?

______ obungi bw’amahabi mumiosi chidatu chibitire_______ NOMBA_______ Sahahasa

_______number of times in past 3 months OR 97. Not sure

*Nikali mbwe yiwumawo esyala nomba ehiiho cholabirira chaliwo, duuma oche hu* ***Esitundu 4***

*If no supervision visits or meetings took place skip to* ***Section 4***

Ehiiho chino ech’endabirira nesyala chali yena?

1. Where did these supervision visits or meetings take place?

Hunderero y’ebyobulamu (fundihira ndala)

- 1. At the health center (*circle one)*

Eeh Haba Sihahasa Ahayire okaluhamo

1. Yes 2. No 97. Not sure 98. Refused to answer

Mulukongo lwange (fundihira ndala)

- 1. In my village (*circle one)*

Eeh Haba Sihahasa Ahayire okaluhamo

1. Yes 2. No 97. Not sure 98. Refused to answer

Ebindi (totolaho)

- 1. Other (describe): _________________________________

Bananu abenyigira mu hiiho ne njihala cholabirira chiwali nacho mu miosi chidatu chibitire ?

1. Who participated in any of the supervision visits or meetings that you had in the past three months?

Basibiri/abahoosi bo munderero (fundihira ndala)

- 1. Staff from the health facility (*circle one)*

Eeh Haba NOMBA Sihahasa Ahayire okaluhamo

1. Yes 2. No OR 97. Not sure 98. Refused to answer

Abeby’obusiroonyo ohutula hu lugala (fundihira ndala)

- 1. District health team (*circle one)*

Eeh Haba NOMBA Sihahasa Ahayire okaluhamo

1. Yes 2. No OR 97. Not sure 98. Refused to answer

Basiribahulihi b’omuluha (fundihira ndala)

- 1. Parish coordinators (*circle one)*

Eeh Haba NOMBA Sihahasa Ahayire okaluhamo

1. Yes 2. No OR 97. Not sure 98. Refused to answer

Basibiri/abahoosi ba Nasietuse (fundihira ndala)

- 1. NGO staff on projects (*circle one)*

Eeh Haba NOMBA Sihahasa Ahayire okaluhamo

1. Yes 2. No OR 97. Not sure 98. Refused to answer

Abataha b’ohusitudu (fundihira ndala)

- 1. Community representative (*circle one)*

Eeh Haba NOMBA Sihahasa Ahayire okaluhamo

1. Yes 2. No OR 97. Not sure 98. Refused to answer

Ebindi (bilambaseho ano) ________________________________________________________________

- 1. Other (*list*): ________________________________________________________________________

Mu ndala hu ngeniya nomba muhiiho ch’ohulabirira chiwali nacho, abali nibewunja emirimo chawo baholaho bilala hubino?

1. During any of the supervision visits or meetings that you had, did your supervisors do any of the following?

Behenenya my lukalala lwange lwa VHT (fundihira ndala)

- 1. Reviewed my VHT register and/or reports (*circle one)*

Eeh Haba NOMBA Sihahasa Ahayire okaluhamo

1. Yes 2. No OR 97. Not sure 98. Refused to answer

Behenenya ohubona nindali nende amaleesi kamala, eby’ohosesa humilimo change (fundihira ndala)

- 1. Checked to see if I had enough medicines, supplies and equipment to do my work (*circle one)*

Eeh Haba NOMBA Sihahasa Ahayire okaluhamo

1. Yes 2. No OR 97. Not sure 98. Refused to answer

Bamberesaho amakaluso akadiira hu maleesi kandakaba abalwaye (koti _ obungi, mubiha, mubanga siina ly’omira)

- 1. Provided feedback on any medicines I gave to clients (eg – dose, duration, how often to take it)

Eeh Haba NOMBA Sihahasa Ahayire okaluhamo

1. Yes 2. No OR 97. Not sure 98. Refused to answer

Ndafuna obuhonyi huhukonyeresa hubukata bwalimo (fundihira ndala)

- 1. Helped me to find solutions to problems (*circle one)*

Eeh Haba NOMBA Sihahasa Ahayire okaluhamo

1. Yes 2. No OR 97. Not sure 98. Refused to answer

Byahoonya ohwongeraho eholegana yange nende abataha musitundu (fundihira ndala)

- 1. Helped me to improve linkages between me and the community (*circle one)*

Eeh Haba NOMBA Sihahasa Ahayire okaluhamo

1. Yes 2. No OR 97. Not sure 98. Refused to answer

Byahoonya ohwongeraho eholegana yange nende enderero (fundihira ndala)

- 1. Helped me to improve linkages between me and the health center (*circle one)*

Eeh Haba NOMBA Sihahasa Ahayire okaluhamo

1. Yes 2. No OR 97. Not sure 98. Refused to answer

Byahoonyah ohweka n’ohumanya amakeesi n’obujusi buyaha (fundihira ndala)

- 1. Helped me to learn new knowledge and skills (*circle one)*

Eeh Haba NOMBA Sihahasa Ahayire okaluhamo

1. Yes 2. No OR 97. Not sure 98. Refused to answer

**ESITUNDU SY’OHUNE (4)**

**SECTION 4**

**Esitundu sino sibusa ebidira hungere yibidiha bikabuhane nga lubibere nibihuhonyaho mumirimo chawo**

**This section collects information about how supportive different groups are for your work**

Mahabi kanga k’obereho nende enomanoma nende mulala hu bohoosi b’enderero mumiosi chidatu chibitire (fundihira ndala)

1. How often have you have had contact (verbal or physical) with a health facility staff member over the past three months? (*circle one)*

Halala nomba ohisawo mu wiki 1 hu 3 mu mwosi Obitabita 1 hu 3 mubyosi Hasiyibechangawo

1. Once or more a week 2. 1 to 3 times a month 3. No more than 1 to 3 times in total 4.Never

Obasa oti enderero yino yoholegananga nayo yihuyambaho muhuhola emirimo chawo koti VHT?

1. Do you feel that the health facility you are attached to is supportive of your work as a VHT?

Eeh Haba

1. Yes 2. No

Nikali mbwe **EEH**, beresaho esyoboneraho mungeri ndala yibabere nibahuyambiramo (sata mubwimbikiti esyoboneraho)

23.1 If **YES**, please give an example of how they have been supportive (*write summary of the example given)*:

______________________________________________________________________________________

_____________________________________________________________________________________

Obasa oti enderero yino yoholegananga nayo yisima handi yebasa emirimo chohaloa?

1. Do you feel that the health facility you are attached to recognizes or respects you for the work you do?

Eeh Haba

1. Yes 2. No

Nikali mbwe **EEH**, beresaho esyoboneraho mungeri ndala yibabere nibasima handi bahwebasa (sata mubwimbikiti esyoboneraho)

24.1 If **YES**, please give an example of how they have recognized and respected you (*write summary of the example given)*:

_______________________________________________________________________________________

________________________________________________________________________________________

Obasa oti ab’ebyobutuki babere bahuhonya mumirimo chawo koti VHT? (fundihira ndala)

1. Do you feel that the local government is supportive of your work as a VHT? (*circle one)*

Eeh Haba

1. Yes 2. No

Nikali mbwe **EEH**, beresaho esyoboneraho mungeri ndala yibabere bahuhonyeramo (sata mubwimbikiti esyoboneraho)

25.1 If **YES**, please give an example of how they have been supportive (*write summary of the example given)*:

______________________________________________________________________________

______________________________________________________________________________

Obasa oti ab’ebyotuki basima n’ohwebasa hulwemirimo chohola?

1. Do you feel that the local government recognizes or respects you for the work you do?

1. Yes 2. No

Nikali mbwe **EEH,** beresaho engeri ndala yobasa oti basima handi bahwebasa (sata mubwimbikiti esyoboneraho)

26.1 If **YES**, please give an example of how they have recognized and respected you (*write summary of the example given)*:

_______________________________________________________________________________

______________________________________________________________________________

Obasa oti abataha besitundu bahuhonyaho mumirimo chawo nga VHT (fundiha ndala)

1. Do you feel that the community is supportive of your work as a VHT? (*circle one)*

Eeh Haba

1. Yes 2. No

Nikali mbwe **EEH**, beresaho engeri ndala yobasa oti bahuhonyerengeho (sata mubwimbikiti esy’obonereho)

27.1 If **YES**, please give an example of how they have been supportive (*write summary of the example given)*:

___________________________________________________________________________________

___________________________________________________________________________________

Obasa oti abataha b’esitundu basima handi bahwebasa emirimo chawo?

1. Do you feel that the community recognizes or respects you for the work you do?

Eeh Haba

1. Yes 2. No

Nikali mbwe **EEH**, beresaho engeri ndala yobasa oti basimiramu handi bebasa (sata mubwimbikiti esy’obonereho)

26.1 If **YES**, please give an example of how they have recognized and respected you (*write summary of the example given)*:

_________________________________________________________________________________________

_________________________________________________________________________________________

Ofunireho eby’ohusima nomba erubia mumiosi chidatu enyuma ohwema nende hu mirimo chawo nga VHT

1. Have you received any allowances or money for your work as a VHT in the past 3 months?

Eeh Haba

1. Yes 2. No

Wafunaho bilala hubino eby’ohwehonyesera mumiosi chidatu chibitireho nga VHT

1. Have you received any of the following benefits in the past 3 months for your work as a VHT?

Ebisolo by’otuka

30.1 Livestock

Eeh Haba

1. Yes 2.No

Esyenanda

30.2 Meals

Eeh Haba

1. Yes 2.No

Eby’ohwehonyera

30.3 Materials

Eeh Haba

1. Yes 2.No

Ohuhusia mumirimo

30.4 Promotions

Eeh Haba

1. Yes 2.No

Ebindi (totolaho)

30.5 Other (*describe)*:

___________________________________________________________________________________

**ESITUNDU SY’OHUTANU (5)**

**SECTION 5**

**Sino situndu sibusa ebidira hu ngeri yiwewuliramo hubidira humirimo chawo**

**This section collects information about how you feel about your work**

Wewulira otye hubidira hubibaso bino? (ndoola homo silala hubibaso bino)

How do you feel about the following statements? (*Tick one response per statement*)

|  | **Fukirira namani**  **Strongly agree (1)** | **Fukirira**  **Agree (2)** | **Sifukirira handi sinyingana**  **Neither agree nor disagree (3)** | **Nyingana**  **Disagree (4)** | **Nyingana muno**  **Strongly disagree (5)** |
| --- | --- | --- | --- | --- | --- |
| Ndesunga ohuba mulala hu VHT  31. I am proud to be a VHT | **** | **** | **** | **** | **** |
| Ndimusangafu hu lw’emirimo cha VHT chihola  32. I feel happy with the VHT work that I do | **** | **** | **** | **** | **** |
| Nyala ndabucha bulayi nende ba VHT bandi  33. I can interact easily with other VHTs | **** | **** | **** | **** | **** |
| Balala bahola nabo bamanyireho hulw’esinani syange mumirimo cha VHT  34. I am known by others I work with for my reliability in my VHT work | **** | **** | **** | **** | **** |
| Ndi nende amakesi nende obujusi buhola emirimo change bulayi  35. I have the knowledge and skills to do my job well | **** | **** | **** | **** | **** |

Wewulira otye hubidira hubibaso bino? (ndoola homo silala hubibaso bino)

How do you feel about the following statements? (*Tick one response per statement*)

|  | **Bulisiha**  **Always (1)** | **Ebiha bihira obungi**  **Most of the time (2)** | **Esiha sindi**  **Sometimes (3)** | **Sitera**  **Rarely (4)** | **Sisiriwo**  **Never (5)** |
| --- | --- | --- | --- | --- | --- |
| Honyangaho ba VHTs bandi mulukongo lwange ohwekaho obujusi buyaha  36. I help other VHTs in my village to learn new skills |  |  |  |  |  |
| Tamu ba VHTs bandi mulukongo lwange ohubakachaniaho muby’ohumaya humuluku  37. I encourage other VHTs in my village to discuss challenges |  |  |  |  |  |
| Ninjekeho obujusi buyaha, mbuta mumirimo change cha VHT  38. When I learn new skills, I apply them in my VHT work |  |  |  |  |  |
| Tusangahoyo esy’ohola nihuba nihuhubachakania ebibaso hubidira humadinyu nende ba VHTs bandi mulukongo  39. I suggest solutions when discussing challenges with other VHTs in my village |  |  |  |  |  |
| Mberesangoho amakaluso kadira huhola yaba VHTs bali mulukonog lwange  40. I give feedback to other VHTs in my village on their performance |  |  |  |  |  |
| Mbechange humulimo paka lumala emirimo change choludaalo  41. I stay on the job until I complete my tasks |  |  |  |  |  |
| Malanga eby’ohoola byange bidira hu VHT mubiha  42. I complete my VHT tasks on time |  |  |  |  |  |
| Ebilonderera bya VHT mbimanyire bulayi  43. The goals of my VHT work are very clear to me |  |  |  |  |  |
| Ndewunjaho muhola yemirimo change ese omwene  44. I evaluate my own work performance |  |  |  |  |  |

Bolaho ebidinyu bihirayo bidatu byonyola nga VHT

45. What are the three greatest challenges that you face as a VHT?

45.1.____________________________________________________________________________________

45.2.____________________________________________________________________________________

45.3.____________________________________________________________________________________

**ESITUNDU SYOMUGANGA (6)**

**SECTION 6**

**Sino situndu sibusa ebidira enjeka nende byomanyireho hubudinyu buli mubana nende amahaye bali asito**

**This section collects information about training and your knowledge of danger signs in children and pregnant women**

Wekaho wola musikanda sina?

1. How many years of school have you completed?

__________ (emyaka)

___________ (years)

Wali hoho mu njeka ya VHT nende etekeha chayo chindi? (fundihira ndala)

1. Have you ever attended the VHT basic training program? (*circle one)*

Eeh Haba

1. Yes 2. No

Nikali mbwe **EEH,**  mumwosi sina nende omwaka kuwamalirimo?

- 1. If **YES**, in which month and year did you complete that training?

________ omwosi ________ omwaka

_________ month _________ year

Wamalirisaho enjeeka yindi nga lwobere noli ohola koti VHT? Eeh Haba

1. Have you ever completed any other training programs while you have been working as a VHT?

1.Yes 2.No

Nikali mbwe **EEH:** fundihira yibichanakana

If YES: *circle those that apply below*

Enjeeka yohumanya ebidira humalwaye n’amachuni k’abaana badoto Eeh Haba

- 1. Integrated community case management of childhood illnesses (ICCM): 1.Yes 2.No

Enjibula y’abaana bayeranu Eeh Haba

- 1. Family planning: 1.Yes 2.No

Wediinge Eeh Haba

- 1. HIV AIDS: 1.Yes 2.No

Omusujja kw’esuuna Eeh Haba

- 1. Malaria: 1.Yes 2.No

Ebindi (totolaho)

- 1. Other (*list):*

_______________________________________________________________________________________

Madinyu siina akali mubahasi bali asito? (fundihira by’omanyire oti VHT yakonyeresa yahahasa)

1. What are the danger signs in pregnant women? (*circle the ones that the VHT correctly identifies but do not read these to the VHT)*

Ohudachirira mubuhasi

48.1 Vaginal bleeding

Ohwasamula

48.2 Fever

Omutwe ohomaka

48.3 Severe headache and blurred vision

Ohufwimba hw’enjala, esura nomba amakulu

48.4 Swelling of fingers, face or legs

Ebifwimba

48.5 Convulsions

Obuchuni asi wenda

48.6 Severe pain in the lower part of the belly

Sataho asi obungi bw’odinyu bu abolereho ____________

48.7 *Write total number of danger signs correctly identified*: ______________

Obudinyu sina buba mu baana bahwebulwa? (fundihiraho bu VHT abolere)

1. What are the danger signs in a newborn? (*circle the ones that the VHT correctly identifies but do not read these to the VHT)*

Obudinyu muhuyera emyuya

49.1 Difficulty in breathing or chest in-drawing

Obudidi mubukali

49.2 Very small in size

Okenda natakala

Obala omubiri nomba kunyita

49.3 Is too hot or too cold

Sanyala ohununa

49.4 Is not able to breastfeed

Olurira ohutula amafira

49.5 Redness around cord, bleeding cord or cord with pus

49.6 Convulsions or abnormal movement

Sataho asi obungi bw’odinyu bu abolereho ____________

49.7 *Write total number of danger signs correctly identified*: ______________

Budinyu sina obwekesa mu baana badoto bahwebulwa? (fundihira by’omanyire oti VHT yakonyeresa yahahasa)

1. What are the danger signs in a child? (*circle the ones that the VHT correctly identifies but do not read these to the VHT)*

Sanyala ohulia

50.1 Unable to feed

Ohusala buli syala

50.2 Vomiting everything

Ohwoma esirifu nende ehololo n’ohwemisa

50.3 Dehydration with cough or fever

Obujongu

50.4 Very weak

Ohudembuha

50.5 Unconscious

Ohuwerereramu

50.6 Convulsions

Amabanga mubunyaka

50.7 Blood in stool

Sataho asi obungi bw’odinyu bu abolereho ____________

50.8 *Write total number of danger sign correctly identified:* ______________

**Webale muno ohuyira ebiha byawo ohukaluhamo mubitebo bino, syoholere husima**

**Thank you for taking the time to answer these questions, your contribution is most appreciated.**
